# Supplementary material for: The role of spectator modes in the quantum-logic spectroscopy of single trapped molecular ions
Source: Commun Phys. 2025 Nov 25;8(1):471. doi: 10.1038/s42005-025-02373-x (PMC12646960; doi:10.1038/s42005-025-02373-x)
Supplement: Supplementary file 1 — Supplementary Information [file 42005_2025_2373_MOESM1_ESM.pdf]

Supplementary Information

The role of spectator modes in the quantum-logic spectroscopy of single trapped molecular ions

Mikolaj Roguski[1], Aleksandr Shlykov[1], Ziv Meir[1,2], Stefan Willitsch[1*]

[1]Department of Chemistry, University of Basel, Basel, Switzerland.
[2]*current address*: Department of Physics of Complex Systems, Weizmann Institute of Science, Rehovot, Israel.

*Corresponding author(s). E-mail(s): stefan.willitsch@unibas.ch;

Supplementary Note 1: Derivation of Eq. (10) from the main text

Note: Equations from the main text are referenced using the same numbering as in the main text. Equations introduced in this Supplementary Information are labelled with an 'S' before their number.

In this section, we outline the derivation of Eq. (10) from Eq. (9). Details on the description of normal modes of a Coulomb crystal of two ions with unequal masses in a harmonic trap can be found elsewhere [1, 2].

Consider the 1D model of a travelling optical lattice interacting with a two-ion string described by the Hamiltonian $\hat{H}$ from Eq. (9). The motion of the ions can be described in terms of two normal modes – in-phase (denoted with '$-$') and out-of-phase (denoted with '$+$') mode, with the frequencies given in Eq. (3).

First, the Hamiltonian from Eq. (9) is separated into time-independent and -dependent parts:

$$\hat{H} = \hat{H}_0 + \hat{H}_I(t), \tag{S1}$$

where

$$\hat{H}_0 = \hbar\omega_-(\hat{a}_-^\dagger \hat{a}_- + \frac{1}{2}) + \hbar\omega_+(\hat{a}_+^\dagger \hat{a}_+ + \frac{1}{2}), \tag{S2}$$

$$\hat{H}_I(t) = \sum_{j=1,2} 2\Delta E_{ac}^{0,j}(1 + \cos(2k\hat{z}_j - \omega_l t)). \tag{S3}$$

The position operators $\hat{z}_j$ for ions $j = 1, 2$ are related to normal mode coordinates $\hat{z}_\pm$ by:

$$\begin{aligned} \hat{z}_1 &= \sqrt{\mu}\,(\hat{z}_+ \cos\theta + \hat{z}_- \sin\theta) + z_1^{init.}, \\ \hat{z}_2 &= -\hat{z}_+ \sin\theta + \hat{z}_- \cos\theta + z_2^{init.}, \end{aligned} \tag{S4}$$

with $z_j^{init.}$ as the equilibrium position of the ions and the angle θ defined as in Eq. (4).

In the interaction picture, the normal-mode position operators become:

$$\begin{aligned} \hat{z}_+ \to \hat{z}'_+(t) &= z^0_+ \left(a^\dagger_+ e^{i\omega_+ t} + a_+ e^{-i\omega_+ t} \right), \\ \hat{z}_- \to \hat{z}'_-(t) &= z^0_- \left(a^\dagger_- e^{i\omega_- t} + a_- e^{-i\omega_- t} \right), \end{aligned} \tag{S5}$$

where $z^0_\pm$ was defined in Eq. (2).

The interaction Hamiltonian in the interaction picture becomes [3]:

$$\begin{aligned} \hat{H}'_I(t) &= \sum_{j=1,2} 2\Delta E^{0,j}_{ac} \left(1 + \cos\left(2k\hat{z}'_j - \omega_l t\right)\right) \\ &\approx \sum_{j=1,2} 2\Delta E^{0,j}_{ac} \left(\cos\left(2k\hat{z}'_j - \omega_l t\right)\right) \\ &= \sum_{j=1,2} \Delta E^{0,j}_{ac} \left(\exp\left(i(2k\hat{z}'_j - \omega_l t)\right) + c.c.\right). \end{aligned} \tag{S6}$$

Here, the constant term in the first line is an energy shift that does not contribute to the dynamics and was thus omitted without loss of generality.

The dynamics of the two-ion crystal interacting with the modulated optical-dipole force is given by the Schrödinger equation:

$$i\hbar\partial_t|\psi_I(t)\rangle = \hat{H}'_I(t)|\psi_I(t)\rangle\,. \tag{S7}$$

The motional wavefunction is defined on the 2D Fock space of two normal modes:

$$|\psi_I(t)\rangle = \sum_{n_+,n_-} C_{n_+,n_-}(t)|n_+,n_-\rangle \tag{S8}$$

with the time-dependent coefficients $C_{n_+,n_-}(t)$.

By combining Eq. (S7) with Eqs. (S6), (S4), (S5) and (S8) one obtains:

$$\begin{aligned} i\hbar\dot{C}_{m_+,m_-}(t) = \sum_{n_+,n_-} & C_{n_+,n_-}\langle m_+,m_-| \left[\Delta E^{0,1}_{ac}\left(\exp\left(2ik(\sqrt{\mu}\left(\hat{z}'_+\cos\theta + \hat{z}'_-\sin\theta\right))\right) - i\omega_l t + i\phi_1\right) + c.c.\right)\right] \\ &+ \left[\Delta E^{0,2}_{ac}\left(\exp\left(2ik((-\hat{z}'_+\sin\theta + \hat{z}'_-\cos\theta))\right) - i\omega_l t + i\phi_2\right) + c.c.\right)\right] |n_+,n_-\rangle\,. \end{aligned} \tag{S9}$$

Here, the initial position of the ions was incorporated into the phase shifts ϕ_j.

To simplify the treatment, three assumptions are made:

1. The target mode can only change by $\pm$ 1 phonon at a time.
2. The spectator mode population is not affected by the ODF pulse.
3. There is no phonon exchange between the modes.

Points 1 and 2 are valid for small detunings of the lattice-modulation frequency from the target-mode frequency, when it is much smaller than the mode frequencies, the lattice frequency and the detuning from the spectator mode, i.e. $|\delta_-| \ll \omega_+, \omega_-, \omega_l, |\delta_+|$.

To simplify the Eq. (S9), we use the relation [4, 5]:

$$\langle n+s|e^{i\,\eta(a+a^\dagger)}|n\rangle = e^{-\eta^2/2}\eta^{|s|}\sqrt{\frac{n_<!}{n_>!}}L^{|s|}_{n_<}(\eta^2). \tag{S10}$$

where s is the change of the number of phonons and $n_{<(>)}$ is the lesser (greater) of n and $n+s$. From assumptions 1 and 2 above, $s = \pm 1$ for the n_- mode and $s = 0$ for the n_+ mode. Other transitions between motional states are neglected.

In the rotating-wave approximation, we thus get:

$$i\hbar\dot{C}_{n_+,n_-}(t) = \sum_{j=1,2} \Delta E^{0,j}_{ac} e^{-(\eta^{(j)}_+)^2/2} L^0_{n_+}((\eta^{(j)}_+)^2) e^{-(\eta^{(j)}_-)^2/2}\eta^{(j)}_-$$

$$\times \left[C_{n_+,n_--1} e^{-i\delta_- t + i\phi_j} \frac{L^1_{n_--1}((\eta_-^{(j)})^2)}{\sqrt{n_-}} \right.$$

$$\left. + C_{n_+,n_-+1} e^{+i\delta_- t - i\phi_j} \frac{L^1_{n_-}((\eta_-^{(j)})^2)}{\sqrt{n_- + 1}} \right], \tag{S11}$$

with Lamb-Dicke parameters defined according to Eqs. (11-14). By rearranging this equation and using the definitions of DW factors from Eq. (5), we finally obtain Eq. (10).

Supplementary References

[1] Morigi, G., Walther, H.: Two-species coulomb chains for quantum information. Eur. Phys. J. D. **13**, 261–269 (2001)

[2] Home, J.P., Hanneke, D., Jost, J.D., Leibfried, D., Wineland, D.J.: Normal modes of trapped ions in the presence of anharmonic trap potentials. New J. Phys. **13**(7), 073026 (2011)

[3] Najafian, K., Meir, Z., Sinhal, M., Willitsch, S.: Identification of molecular quantum states using phase-sensitive forces. Nat. Comm. **11**(1), 4470 (2020)

[4] Wineland, D.J., Itano, W.M.: Laser cooling of atoms. Phys. Rev. A **20**(4), 1521 (1979)

[5] Leibfried, D., Blatt, R., Monroe, C., Wineland, D.: Quantum dynamics of single trapped ions. Rev. Mod. Phys. **75**(1), 281 (2003)
